# Male National Basketball Association G-League and Collegiate Basketball Athletes Have a High Prevalence of Radiographic Ankle Abnormalities

**DOI:** 10.1016/j.asmr.2024.100980

**Published:** 2024-07-26

**Authors:** Krishna Mandalia, Ryan Harrington, Albert Mousad, Bryan Jenkin, Katharine Ives, Sarav Shah

**Affiliations:** a Tufts University School of Medicine, Boston, Massachusetts, U.S.A.; b New England Baptist Hospital, Boston, Massachusetts, U.S.A.; c New England Shoulder and Elbow Center, Boston, Massachusetts, U.S.A.

## Abstract

**Purpose:**

To characterize radiographic foot/ankle bony abnormalities in elite-level, asymptomatic male basketball athletes and to investigate the association between anthropometric (age, height, weight) or sport-specific characteristics (total exposures, player position, pregame ankle taping) and the prevalence of abnormal radiographic findings in asymptomatic basketball athletes.

**Methods:**

Elite-level basketball players who underwent routine, preseason static radiographic imaging, including anteroposterior, lateral, and mortise views of the ankle were included. Radiographs were collected from asymptomatic athletes participating in preseason history and physical with negative anterior drawer/talar tilt test. Radiographs were evaluated by a musculoskeletal radiologist and board-certified orthopaedic surgeon; kappa statistics were used to evaluate agreement.

**Results:**

Fifty-four basketball players (34 collegiate, 20 professional; mean age 21.5 years) were included, totaling 5,148 player exposures from 2017 to 2019. In total, 106 ankles presented with radiographic findings (98.15%). The most prevalent radiographic finding was pes planus (47.22%), followed by degenerative joint disease (DJD; 33.33%), talonavicular sclerosis (28.70%), prominent stieda process (25.93%), os trigonum (20.93%), os subfibulare (11.11%), pes cavus (5.56%), subtalar coalition (2.78%), and cavovarus (0.93%). Height ≥80 inches was significantly associated with talonavicular sclerosis and Kellgren-Lawrence 1 changes.

**Conclusions:**

This study showed a strong association between height and talonavicular sclerosis and DJD, as well as a relatively high prevalence of pes planus and DJD in asymptomatic collegiate and professional basketball players.

**Level of Evidence:**

Level II; Cross-sectional study.

An estimated 450 million individuals participate in basketball worldwide, including both professional and recreational settings.^1^, ^2^, ^3^ Across all levels of basketball participation, ankle injuries are the most common injury reported for both sexes.^1^, ^2^, ^3^, ^4^, ^5^, ^6^, ^7^Within the National Basketball Association (NBA), the ankle remains the most frequently injured joint, and morbidity related to these ankle injuries accounts for the second most common cause of missed games.^2^^,^^8^^,^^9^ High-level basketball athletes frequently are exposed to lower-limb stress.^10^ It has been shown in soccer athletes, and postulated in basketball athletes, that lower-limb stresses may be an etiology of radiographic ankle-foot joint abnormalities, including early degenerative joint disease (DJD).^11^^,^^12^

Historically, abnormal imaging findings have been shown to increase the relative risk of certain orthopaedic injuries in asymptomatic individuals’ ankle, rotator cuff, hip, labrum, and cervical/lumbar spinal joints.^13^, ^14^, ^15^, ^16^, ^17^, ^18^, ^19^ Certain ankle radiographic findings are relatively common in basketball athletes, given that anatomic variants of the ankle-foot joint complex (i.e., accessory ossicles and sesamoid bones) are commonly found in the general population, although it is unknown whether these radiographic findings are prevalent at a rate differing than that of the general population.^20^ These anatomic variants typically are not clinically significant; however, in the setting of sports injury/trauma, they may be misdiagnosed and lead to overtreatment.^21^

Given the overuse and high-impact physical activity associated with basketball training and competitive play and that previous biomechanical studies have shown an association between larger mass moments of inertia and the risk of lateral ankle injuries, it is imperative that clinicians are able to distinguish between lesions that present asymptomatically at a greater prevalence in basketball athletes from lesions that may indicate abnormal pathophysiology of the foot-ankle complex.^22^ Furthermore, it is imperative to identify and counsel athletes who may be associated with greater rates of less commonly investigated radiographic abnormalities (i.e., talonavicular sclerosis, pes planus, and DJD). These may remain asymptomatic or, with the continued physical stress that elite basketball play demands, contribute to issues of biomechanical instability or symptomatic pathophysiology of the foot-ankle complex. The purposes of this study were to characterize radiographic foot/ankle bony abnormalities in elite-level, asymptomatic male basketball athletes and to investigate the association between anthropometric (age, height, weight) or sport-specific characteristics (total exposures, player position, pregame ankle taping) and the prevalence of abnormal radiographic findings in asymptomatic basketball athletes. We hypothesized that height would be associated with a greater prevalence of radiographic abnormalities.

## Methods

This study was approved by the New England Baptist Hospital Institutional Review Board and the NBA Team Physicians Research Committee. Patient data were collected during preseason evaluation of NBA G-League (Maine Celtics) and Men’s Division I (Merrimack College) and Division III (Tufts University) basketball players between 2017 and 2019. Patients were excluded if they had a diagnosis of ligamentous laxity or sustained an ankle injury outside of basketball-related activity. In keeping with established protocol, each player was evaluated anew each preseason.

### Imaging

Static radiographic imaging was collected as part of routine preseason protocols, including anteroposterior, lateral, and mortise views of the ankle. Radiographs were collected from asymptomatic athletes participating in preseason workouts with a negative anterior drawer and talar tilt test. Of note, talar tilt incidence was not investigated, as stress ankle imaging was not available for review. Radiographic imagining was evaluated separately by a dual-fellowship-trained orthopaedic surgeon (S.S.) and a musculoskeletal fellowship-trained radiologist (R.H.). The following specific parameters were chosen to be investigated on the basis of previous literature:

I. Talonavicular changes/sclerosis
  • Deformities in the calcaneopedal unit may lead to abnormal foot mechanics, such as atypical loading and unloading of the foot.^23^
II. Medial clear space widening
  • Medial clear space width is correlated with deltoid ligament stability.^24^^,^^25^
III. Os subfibulare
  • Studies suggest that an os subfibulare represents an avulsion fracture that may be associated with laxity of the anterior talo-fibular ligament, rather than being a normal anatomic variant.^26^
  • Mild sclerosis was defined as articular joint space narrowing without significant articular destruction, whereas moderate sclerosis was defined as articular destruction, and severe sclerosis was defined as bony ankylosis.
IV. Subtalar coalition
  • There is literature linking subtalar coalition to an increased risk of ankle sprain in athletes. This is postulated to be secondary to restricted movement of the foot complex, leading to increased ligamentous strain during trauma.^27^^,^^28^
V. Cavovarus
  • Cavovarus has been correlated with inversion ankle sprains^29^ and recurrent lateral ankle instability.^30^^,^^31^
VI. Pes planus
  • Pes planus has been associated previously with overuse injuries, such as stress fractures, iliotibial band syndrome, patellofemoral syndromes, Achilles tendinitis, and periostitis, in military trainees.^32^
VII. Tibiotalar malalignment
  • Tibiotalar malalignment has been correlated with supramalleolar varus and valgus deformities, and may be indicative of asymmetric osteoarthritis of the ankle mortise.^33^
VIII. Degenerative joint disease (DJD)
• Early-onset DJD may occur in basketball players, as they are frequently exposed to lower-extremity stress.^11^^,^^12^
IX. Os trigonum
• Presence of an os trigonum may represent failed fusion of a secondary ossification center in the talar body and often is associated with posterior ankle impingement resulting from acute fractures, chronic injury, or microtrauma.^34^
X. Prominent stieda process
  • Presence of a prominent stieda process has been associated with posterior ankle impingement, and stieda process fractures are reportedly common in athletes.^35^

### Statistical Analyses

Two raters (S.S. R.H.) separately evaluated radiographic imaging generated in the study. Scoring for the aforementioned parameters is detailed in Table 1.^1^^,^^32^^,^^36^ Cohen kappa statistics were used to assess interrater agreement and reliability for each measured parameter for each ankle.

**Table 1:** Scoring Parameters for Ankle Radiograph Review

Parameter	Scoring and Notation Method
Talonavicular changes/sclerosis	
Grade 0	Absent
Grade 1	Mild
Grade 2	Moderate
Grade 3	Severe
Medial clear space widening	
Absent widening (0-4)	0
Widening present^32^ (4+)	1
Presence of os subfibulare	
0	Absent
1	Present
Subtalar coalition	
Medial facet	1–3 (1 = good visualization of the radiolucent middle facet joint space with clearly visible subchondral bone cortex of the talar and calcaneal facets, 2 = partial visualization of the radiolucent joint space and subchondral cortex, 3 = no visualization of radiolucent joint space or subchondral cortex).^1^Absent middle facet sign had a sensitivity, specificity, and accuracy of 75%, 98%, and 90%, respectively, for the diagnosis of subtalar joint coalition.^36^
C sign	C sign (1 = normal talus, 2 = partial C-shaped radiopaque arc from the talar dome to the sustentaculum tali, 3 = complete C-shaped radiopaque arc from the talar dome to the sustentaculum tali).Sensitivity, specificity, and accuracy: 75%, 98%, 90% respectively.^36^
Together (absent middle facet sign + positive C sign)	Sensitivity, specificity, and accuracy of 84%, 98%, and 94%, respectively.^36^
Cavovarus	Pes cavus is defined by its appearance in the sagittal (lateral) plane.^1^
1. Calcaneal inclination angle (CIA, also known as calcaneal “pitch” angle).	Grade 0 = angle of 25° ± 5°(normal) Grade 1 = >30° Pes cavus
2. Lateral TC angle (for potential subclassification)	Grade 0 = 35° to 50° (normal) Grade 1 = <35°
Pes planus	Calcaneal inclination angle (CIA, also known as calcaneal “pitch” angle)
Grade 0	Angle of 25° ± 5°(normal)
Grade 1	Angle <20°
Tibiotalar malalignment	
Grade 0	Normal
Grade 1	Abnormal (if >0°, mortise view)
Degenerative joint disease (DJD), graded using the Kellgren-Lawrence system as used in previous studies^34^	
Grade 0	No radiographic features of DJD are present
Grade 1-2	Doubtful joint space narrowing (JSN) and possible osteophytic lipping Or definite osteophytes and possible JSN on anteroposterior weight bearing radiographs
Grade 3-4	Multiple osteophytes, definite JSN, sclerosis, and possible bony deformity Or large osteophytes, marked JSN, severe sclerosis, and definite bony deformity
Presence of os trigonum	
0	Absent
1	Present
Prominent stieda process	
0	Absent
1	Present

TC, talocalcaneal.

Data were stored in a custom Excel spreadsheet (Microsoft, Redmond, WA) and were subsequently analyzed using SPSS Statistical Package, Version 29.0 (IBM Corp., Armonk, NY). The results were considered statistically significant at *P* < .05. The prevalence was reported as a percentage of the total sample of ankles. Anthropometric measurements (age, height, and weight) as well as sport-specific variables (total exposures, player position, and pregame ankle taping) were investigated for their effect on the prevalence of radiographic abnormalities via multiple logistic regression analysis. Pearson correlation and T-distribution analysis was conducted to determine the significance between anthropometric as well as sport-specific variables and total radiographic abnormalities.

## Results

A total of 54 basketball players (34 collegiate, 20 professional) with a mean age of 21.5 ± 2.5 years met inclusion criteria, totaling 5,148 player exposures from 2017 to 2019. Thirty-three (61.1%) of these basketball athletes were classified as backcourt players (point guard or shooting guard), and 11 (20.4%) used pregame ankle taping. Player demographics are showed in Table 2. One-hundred-eight different ankles with corresponding radiographic imaging were reviewed, of which 106 ankles presented with radiographic abnormalities (98.15%). A full summary of scoring parameters, prevalence, and abnormal radiographic findings are found in Table 3.

**Table 2:** Player Demographics

Age, yr, mean	21.5 ± 2.5
Height, inches, mean	76.8 ± 2.9
Weight, lb., mean	205.9 ± 23.9
Player exposures, mean	95.3 ± 28.2
Pregame ankle taping, n (%)	11 (20.4)
Backcourt player, n (%)	33 (61.1)
Collegiate athlete, n (%)	34 (63.0)

**Table 3:** Summary of Scoring, Prevalence, and Abnormal Radiographic Findings

	Talonavicular Sclerosis (0-3)				Medial Clear Space Widening (0-1)		Os Subfibulare(0-1)		Medial Facet(1-3)			C Sign(1-3)		
	0	1	2	3	0	1	0	1	1	2	3	1	2	3
N	77	25	4	2	108	0	96	10.5	97.5	10.5	0	84.5	20.5	3.5
Prevalence(%)	71.3	23.15	3.7	1.85	108	0	88.89	9.72	90.28	9.72	0	78.24	18.98	3.24
% Abnormal	28.70%				0%		9.72%		Subtalar coalition (3.24%)					

	Tibiotalar Malalignment (0-1)		Calcaneal Inclination Angle (–1 to 1)			Lateral TC Angle (0-1)		Kellgren-Lawrence System (0, 1-2, 3-4)			Os Trigonum (0-1)		Prominent Stieda Process (0-1)	
	0	1	–1	0	1	0	1	0	1-2	3-4	0	1	0	1
N	108	0	51.5	51	5.5	77.5	30	72.5	29	6.5	86.5	27	80	29
Prevalence (%)	108	0	47.69	47.22	5.09	71.76	27.78	67.13	26.85	6.02	80.09	25	74.07	26.85
% Abnormal	0%		Pes planus (47.69%); pes cavus (5.09%)			Cavovarus (0.93%)		DJD changes (32.87%)			25%		26.85%	

DJD, Degenerative joint disease.

**Fig 1:**
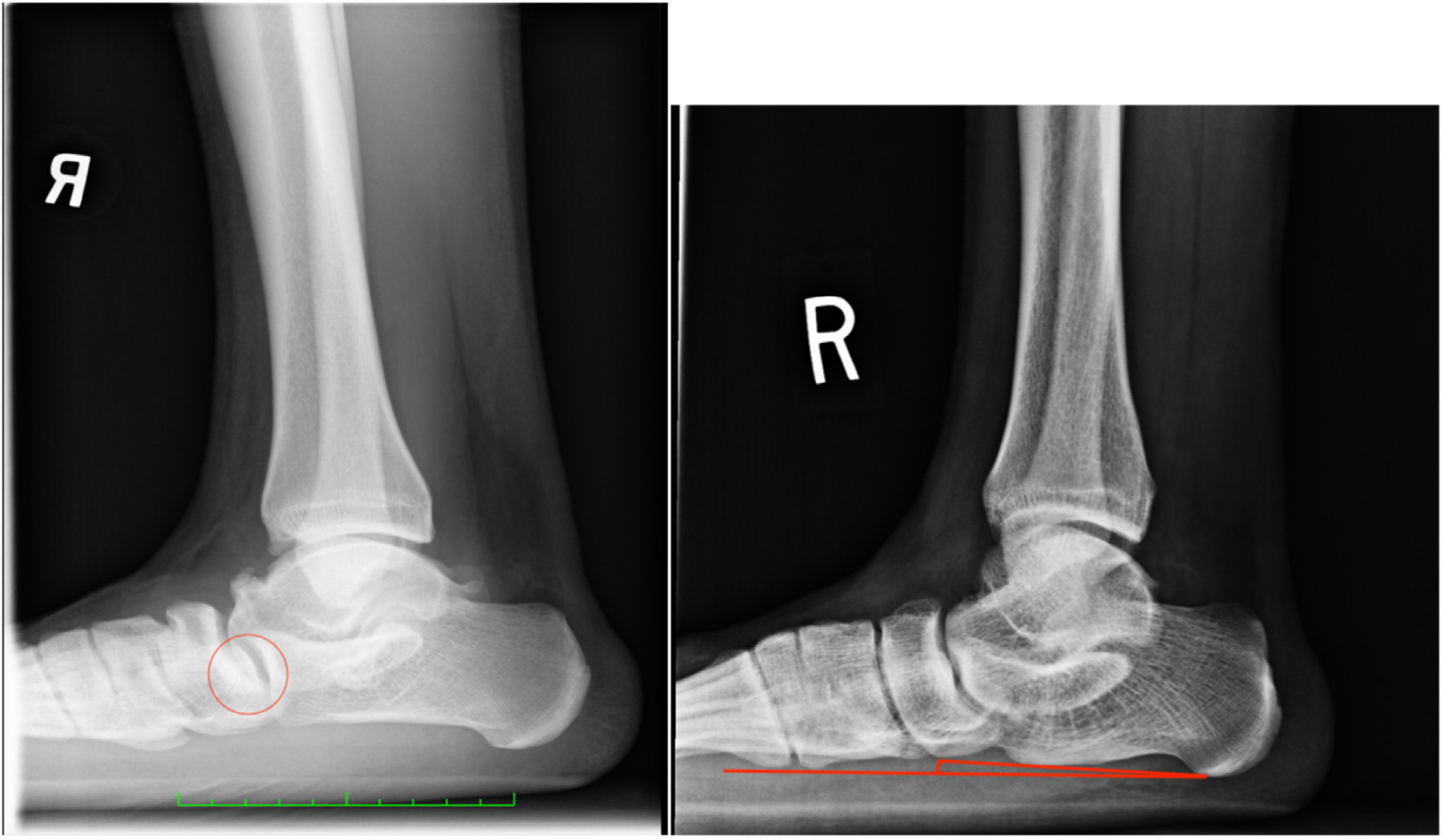
Talonavicular sclerosis/changes (left) and pes planus (left and right). Both are right ankle, lateral radiographs.

**Fig 2:**
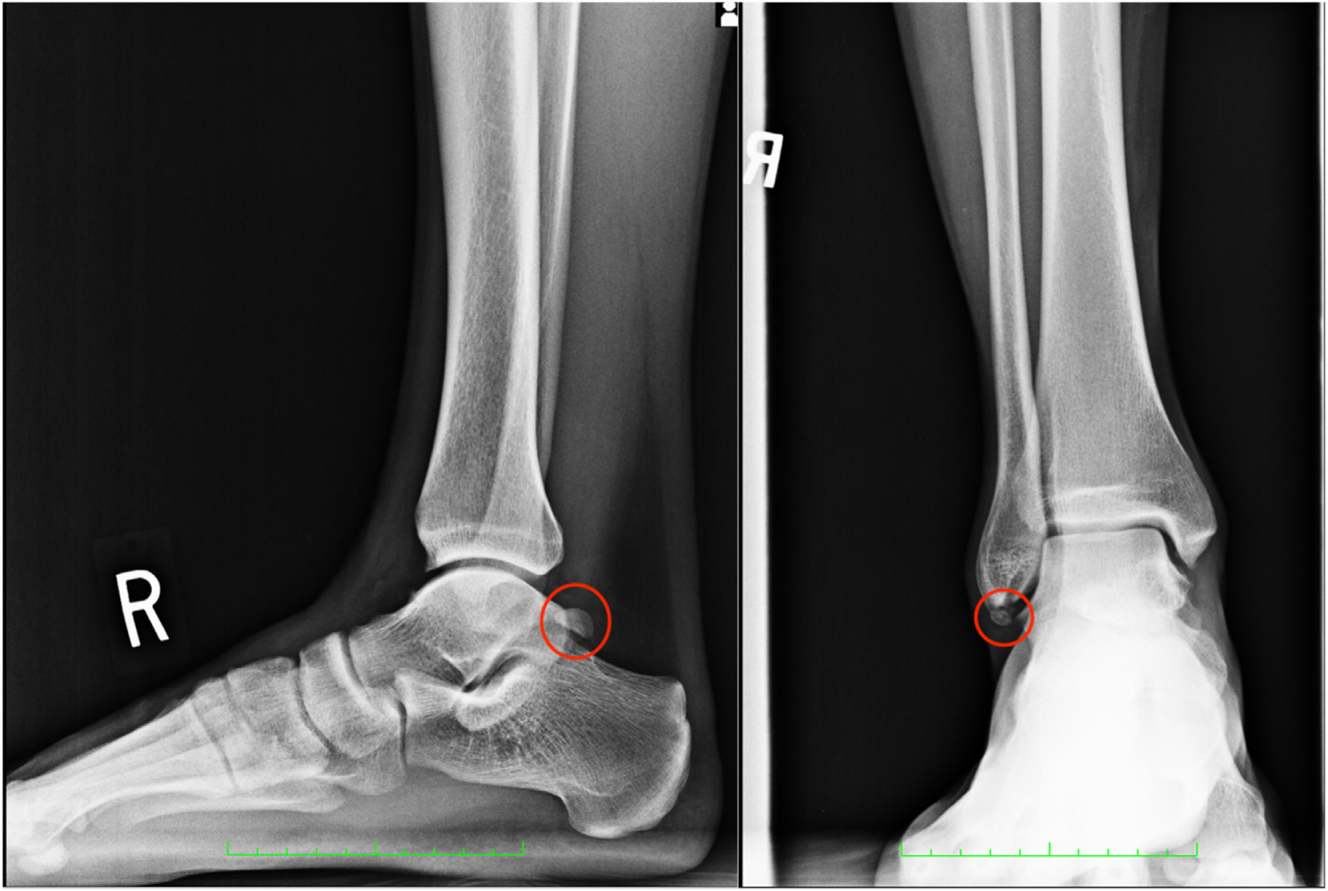
Os trigonum (left) and os subfibulare (right). The left image is a right ankle, lateral radiograph. The right image is a right ankle, anteroposterior radiograph.

Talonavicular changes/sclerosis (Fig 1) was found in 28.7% of radiographs, whereas the prevalence of DJD changes (Kellgren-Lawrence [KL] system) was 33.33%. Subtalar coalition was found in 2.78% of ankles, as denoted by the presence of a full C-sign. No ankles showed an absence of the medial facet. Pes planus (Fig 1) was prevalent in 47.22% of ankles, whereas pes cavus was found in 5.56% of ankles. Of the pes cavus ankles, only one ankle was found to have cavovarus (0.93%) on the basis of concomitant lateral TC angle measurement. Os trigonum (Fig 2) was prevalent in 20.37% of ankles, similarly to the prevalence of a prominent stieda process, which was 25.93%. The prevalence of an os subfibulare (Fig 2) was 11.11%. No ankles showed medial clear space widening nor tibiotalar malalignment.

On regression analysis, a significant inverse relationship was found between height and the absence of DJD changes (KL= 0), to which taller athletes were less likely to present without DJD changes (*P* < .001, odds ratio [OR] 0.071, 95% confidence interval 0.041-0.123). On further analysis, height greater than 6’8” (80”) was significantly predictive of mild (grade 1) talonavicular sclerosis (*P* = .022, OR 10.86). On χ^2^ analysis, height greater than 80” was also significantly associated with KL 1 DJD changes (*P* = .017) and brace use (*P* < .001). Weight was found to be a significant predictor of the presence of os trigonum (OR 1.135, *P* = .025), whereas pregame ankle taping was significantly predictive of the absence of os trigonum (OR 0.031, *P* = .046). Age (Pearson coefficient 0.05, *P* = .73), height (Pearson coefficient = 0.10, *P* = .47), weight (Pearson coefficient = 0.26, *P* = .06), previous injury (Pearson coefficient = 0.12, *P* = .39), and total exposures (Pearson coefficient = 0.55) were not found to be significantly correlated with total radiographic abnormalities on Pearson correlation and T-distribution analysis. No other anthropometric or sport-specific factors were found to be significantly correlated with individual or cumulative radiographic abnormalities.

Regarding inter-rater reliability, 22 individual Cohen kappa analyses were conducted, of which 4 measurements were considered “strong” and the remaining 18 measurements were considered “almost perfect.”^37^ All kappa statistics were statistically significant (*P* < .05). Details of each kappa analysis for the right and left ankle can be found in Appendix Table 1, available at www.arthroscopyjournal.org.

## Discussion

In this study, we found a relatively high prevalence of pes planus, DJD, and talonavicular sclerosis in asymptomatic, collegiate, and professional male basketball players. Few previous studies have comprehensively evaluated these 11 foot and ankle parameters. Talonavicular sclerosis has been suggested to play a role in tensile force transmission during the windlass mechanism in gait.^38^ Furthermore, significant relationships have been found between pes planus and number of injuries sustained in cadets at West Point.^39^ Studies have also shown multisegmental motion in patients with ankle DJD demonstrates significant changes in foot mechanics characterized by altered segment kinematics and a significant reduction in dynamic range of motion at the tibia, hindfoot, forefoot, and hallux when compared with controls.^40^ These findings may serve as valuable data to identify and counsel athletes who may be associated with greater rates of radiographic abnormalities. Furthermore, as physicians evaluating high-level athletes with acute injuries to the foot and ankle, it is critical to understand that these lesions are often present and often asymptomatic. We must be prudent to take into account the full clinical picture and recognize that these findings may or may not be contributing to the problem at hand. Further research is warranted to evaluate how these observed findings specifically play a role in basketball performance and rehabilitation, especially after lateral ankle sprains.

Interestingly, our study reported players taller than 80” were significantly associated with talonavicular sclerosis, with an overall prevalence of 28.7%. This is of particular interest, as talonavicular sclerosis has been associated with degenerative changes of the ankle, chronic stress, and abnormal movement of the foot-ankle complex.^20^^,^^41^ Ankle sprains affect approximately 26% of NBA players on average each season and account for a large number of missed NBA games.^2^ The talonavicular ligament is commonly injured in lateral ankle sprains, and it is therefore conceivable that lateral ankle sprains contribute to talonavicular ligament incompetence and ultimately talonavicular joint sclerosis.^38^ Furthermore, literature suggests a history of ankle sprains is associated with radiographic talonavicular arthritis.^38^^,^^40^^,^^42^ In one risk factor analysis of lateral ankle sprains in military recruits, taller and heavier individuals showed increased lateral ankle sprain morbidity and a greater incidence of lateral ankle sprains, given their larger mass moments of inertia.^22^ To that matter, both talonavicular sclerosis and DJD changes (KL system) have been reported at a high prevalence in those with persistent complaints after lateral ankle sprains.^41^ Thus, the presence of both DJD and talonavicular sclerosis in asymptomatic athletes may be suggestive of a more difficult rehabilitation after lateral ankle sprains.

Furthermore, we found players taller than 80” were significantly associated with KL changes 1 or greater. In the general population, the prevalence of radiographic DJD in the foot-ankle complex has been reported to be as high as 12%,^43^^,^^44^ whereas the prevalence of osteoarthritis and other DJD changes in former elite soccer players has been reported to be between 12% and 17%.^44^ In contrast, our study showed that the prevalence of DJD changes in our elite basketball players was 33%. This suggests that the rigors endured by basketball players may be associated with a greater prevalence of degenerative changes (total: 33%; KL 1-2 = 27%; KL 3-4 = 6%) of the foot-ankle complex as compared with both the general population and elite athletes of other sports. Further investigation is warranted on the significance of radiographic DJD and talonavicular sclerosis and its correlation to clinical osteoarthritis in the long-term. In the short term, further investigation into the significance of these findings and their influence on the rehabilitation of ankle sprains is warranted. Findings such as medial clear space widening and tibiotalar malignment are typically seen in acute injuries and are therefore not seen in our asymptomatic cohort.

Pes planus, or “flat feet,” is a relatively common foot deformity in the general population, with a reported prevalence of 20% to 37%.^36^ Pes planus specifically refers to the loss of the medial longitudinal arch of the foot, which functions as an adaptive support base for the body as well as a means to dissipate weight-bearing forces.^36^ Compared with the general population, our study found the prevalence of pes planus in asymptomatic elite basketball players to be high at 47.22%. This is particularly salient, as the presence of pes planus has been associated with reduced biomechanical stability of the foot-ankle complex in conjunction to reduced body balance and performance on certain athletic parameters. Specifically, an increasing degree of pes planus has been associated with reduced postural control and vertical jump height.^45^ Given the negative biomechanical implications of pes planus, it has also been associated with an increased risk of pain and injury of the lower extremities and lumbar spine.^45^ Levy et al.^39^ found that both flatter and larger feet were significantly associated with a greater incidence of lower-extremity injuries. This is particularly interesting, given that those with larger feet are conceivably taller and/or heavier individuals, which, as previously discussed, is an independent risk factor for increased ankle sprain morbidity, and was studied in a similar population of army recruits.^22^ This may appear paradoxical, as elite-level basketball play demands substantial jump height and postural stability. To that matter, it is important to acknowledge the high level of concomitant neuromuscular training, strength, and conditioning that provides a mechanism of correction to the biomechanic instability created by pes planus. In conjunction, pes planus in these athletes may likely be acquired, as previous studies have showed the acquisition of pes planus in high-impact sports (i.e., basketball) through repetitive stress to the plantar fascia and degenerative changes to the foot-ankle complex.^44^^,^^46^ Although frequently asymptomatic, as in the case of our study, the high prevalence of pes planus in elite basketball players should alert clinicians of altered lower-extremity biomechanics.

The high prevalence of asymptomatic ankle and foot abnormalities showed by our study (98.15%) is comparable with previous literature of elite athletes in other sports investigating the prevalence of asymptomatic ankle and foot abnormalities. In asymptomatic, professional snowboarders (mean age 23.4 years), there was a 100% prevalence of osteochondral and soft-tissue damage of the ankle on magnetic resonance imaging^47^ as well as a high prevalence of asymptomatic abnormalities of the Achilles tendon in elite fencers.^48^ Furthermore, the MRI scans of 86.5% of professional male soccer players showed DJD changes and cartilage lesions of varying severity.^11^ As we evaluate high-level athletes with acute injuries to the foot and ankle, it is critical to understand that these lesions are often present, and we must consider the full clinical picture and recognize that these findings may or may not be contributing to the issue at hand. It is our hope that this study provides clinicians and athletic trainers with information to better guide the diagnosis and treatment of asymptomatic, abnormal radiographic findings of the foot-ankle complex in high-level basketball players. Further studies are warranted to better define the clinical correlations and implications of these radiographic findings.

### Limitations

The primary limitation of this study is that it lacks a control group. Although our study found no association between previous injury and radiographic findings, this may be related to under-reporting and/or recall bias, as previous injury was defined as recall from the athlete. Our cohort of relatively young mean age and high-level male basketball athletes suggest our findings may not be generalizable to the general adult population. Moreover, we did not determine the prevalence of lower-limb malalignment, which may influence the presence of these radiographic abnormalities. In addition, no biomechanical assessments were conducted to analyze the relationship between muscular balance of the lower extremities. This study employed the KL system to evaluate DJD changes; however, other scales/systems exist to determine the degree of DJD in the foot ankle (i.e., Takakura scale).

## Conclusions

This study showed a strong association between height and talonavicular sclerosis and DJD, as well as a relatively high prevalence of pes planus and DJD in asymptomatic collegiate and professional basketball players.

## Disclosures

The authors declare the following financial interests/personal relationships which may be considered as potential competing interests: R.H. reports a relationship with Vertex that includes employment and equity or stocks. S.S. reports a relationship with American Academy of Orthopaedic Surgeons, American Orthopaedic Society for Sports Medicine, and Arthroscopy Association of North America that includes board membership and a relationship with Exactech Inc that includes consulting or advisory. All other authors (K.M., A.M., B.J., K.I.) declare that they have no known competing financial interests or personal relationships that could have appeared to influence the work reported in this paper.
