# Supplementary material for: Male National Basketball Association G-League and Collegiate Basketball Athletes Have a High Prevalence of Radiographic Ankle Abnormalities
Source: Arthrosc Sports Med Rehabil. 2024 Jul 26;6(6):100980. doi: 10.1016/j.asmr.2024.100980 (PMC11701938; doi:10.1016/j.asmr.2024.100980)
Supplement: Appendix Table 1 [file mmc1.docx]

**Appendix Table 1.** Cohen’s Kappa Statistics Summary for Ankle Measurements

| **Right Ankle** | | | | | | | | | | | |
| --- | --- | --- | --- | --- | --- | --- | --- | --- | --- | --- | --- |
|  | **Talonavicular Sclerosis** | **Medial Clear Space Widening** | **Os Subfibulare** | **Medial Facet** | **C Sign** | **Tibiotalar Malalignment** | **Calcaneal Inclination Angle** | **Lateral TC Angle** | **Kellgren-Lawrence** | **Os Trigonum** | **Prominent Stieda Process** |
| **Kappa** | 0.869 | 1 | 0.836 | 0.913 | 0.838 | 1 | 1 | 0.775 | 0.958 | 1 | 0.951 |
| **P-Value** | <0.001 | <0.001 | <0.001 | <0.001 | <0.001 | <0.001 | <0.001 | <0.001 | <0.001 | <0.001 | <0.001 |
| **Left Ankle** | | | | | | | | | | | |
|  | **Talonavicular Sclerosis** | **Medial Clear Space Widening** | **Os Subfibulare** | **Medial Facet** | **C Sign** | **Tibiotalar Malalignment** | **Calcaneal Inclination Angle** | **Lateral TC Angle** | **Kellgren-Lawrence** | **Os Trigonum** | **Prominent Stieda Process** |
| **Kappa** | 0.876 | 1 | 0.78 | 0.731 | 0.897 | 1 | 0.864 | 0.724 | 0.814 | 0.904 | 0.859 |
| **P-Value** | <0.001 | <0.001 | <0.001 | <0.001 | <0.001 | <0.001 | <0.001 | <0.001 | <0.001 | <0.001 | <0.001 |
